# The Extract of the Endophytic Fungus *Penicillium compactum* Inhibits the Replication of Human Coronavirus

**DOI:** 10.3390/ijms27031183

**Published:** 2026-01-24

**Authors:** Jihun Choi, Siyun Lee, Chunghyeon Lee, Seungju Cho, Woochul Jung, Jayhyun Park, Yeong-Geun Lee, Youngae Jung, Chun-Zhi Jin, Hyung-Gwan Lee, Chang Soo Lee, Junsoo Park

**Affiliations:** 1 Division of Biological Science and Technology, Yonsei University, Wonju 26493, Republic of Korea; 2 R&D Team, Korea Mine Rehabilitation and Mineral Resources Corporation, Wonju 26464, Republic of Korea; 3 Integrated Metabolomics Research Group, Western Seoul Center, Korea Basic Science Institute, Seoul 03759, Republic of Korea; 4 Cell Factory Research Center, Korea Research Institute of Bioscience and Biotechnology (KRIBB), 125 Gwahak-ro, Yuseong-gu, Daejeon 34141, Republic of Korea; 5 Fungi Research Division, Nakdonggang National Institute of Biological Resources, Sangju 37242, Republic of Korea

**Keywords:** human coronavirus, *Penicillium compactum*, antiviral, natural compound

## Abstract

Fungal extracts have been reported to exert diverse biological activities, including anti-inflammatory, antibacterial, and antiviral effects. However, the anti-coronaviral properties of fungal extracts remain largely unexplored. In this study, we demonstrated that the *Penicillium compactum extract* (PCE) inhibits the replication of human coronavirus. RD cells were infected with human coronavirus and subsequently treated with PCE. PCE treatment reduced the expression of viral proteins and ameliorated virus-induced cytopathic effects. In addition, PCE markedly decreased viral RNA levels in both the cells and the conditioned medium. Finally, we confirmed that PCE treatment reduced the production of infectious viral particles. Collectively, these findings indicate that PCE exhibits potent antiviral activity against human coronavirus.

## 1. Introduction

Coronaviruses are associated with a wide range of diseases, from the mild common cold to severe and sometimes fatal illnesses [1,2]. Although the recent emergence of bat-derived coronaviruses in humans led to the COVID-19 pandemic, several human coronaviruses—such as HCoV-OC43, HCoV-229E, HCoV-NL63, and HCoV-HKU1—have circulated long before the COVID-19 era [3,4]. Coronavirus infections also affect not only humans but also pet and domesticated animals, including dogs, cats, and pigs [5,6]. Although antiviral agents and vaccines against COVID-19 have been developed, resistant coronavirus variants capable of evading vaccine-induced immunity and antiviral drugs continue to emerge [7,8,9,10,11]. Therefore, alternative therapeutic strategies are urgently needed to prepare for the future emergence of resistant strains.

Fungi have long served as valuable sources of pharmaceutical compounds, producing a wide range of antimicrobial, anticancer, antidiabetic, and anti-inflammatory agents [12]. Classic examples include penicillin, isolated from *Penicillium notatum*, and cephalosporins, derived from *Penicillium cephalosporium* [13,14]. Cyclosporine, an immunosuppressive drug, was obtained from the filamentous fungus *Tolypocladium inflatum* [15], and taxol, a well-known anticancer agent, was isolated from *Taxomyces andreanae* [16]. Polysaccharides from *Ganoderma lucidum* and *Phellinus linteus* have also been reported to exert hypoglycemic effects, contributing to the alleviation of diabetic symptoms [17,18].

Fungal extracts and their metabolites are likewise recognized for their antiviral properties [19,20]. The methanol extract of *Ganoderma lucidum* inhibits HIV-1 replication and HIV-1 protease activity [21,22]. Triterpenoids isolated from *Ganoderma lingzhi* exhibit inhibitory activity against influenza neuraminidase, which is a key enzyme in influenza virus replication [23,24].

More recently, fungi have been investigated for their potential activity against coronaviruses, including SARS-CoV-2. Endophytic fungi isolated from *Artemisia vulgaris* were shown to inhibit the SARS-CoV-2 3CL protease, an essential viral enzyme [25]. In addition, in silico analyses have suggested that compounds present in edible mushrooms may inhibit SARS-CoV-2 3CL protease [26]. Furthermore, extracts of *Aspergillus flavus* demonstrated inhibitory activity against the replication of human coronavirus HCoV-229E [27].

In this study, we sought to identify fungi capable of suppressing coronavirus replication and found that the extract of *Penicillium compactum* (PCE) effectively inhibits human coronavirus replication. We further demonstrated that PCE treatment attenuates coronavirus-induced cytotoxicity and reduces the production of infectious viral particles. These findings highlight the potential of fungi and their derivatives as sources of novel antiviral agents.

## 2. Results

### 2.1. Penicillium Compactum Extract Decreases the Expression of Coronavirus Proteins

**Figure 1:**
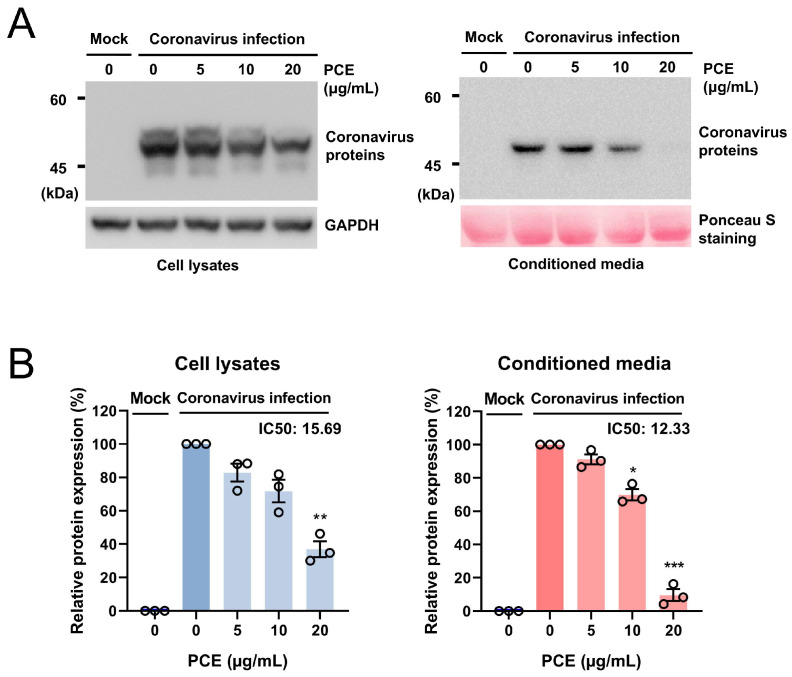
*Penicillium compactum* extract (PCE) reduces the expression of coronavirus proteins. (**A**) PCE decreased the expression of human coronavirus (HCoV-OC43) proteins in a dose-dependent manner. RD cells were infected with HCoV-OC43 and subsequently treated with the indicated concentrations of PCE. At 72 h post-infection, cell lysates and conditioned media were collected, and viral protein levels were assessed by Western blot analysis. (**B**) Western blot bands were quantified and plotted. Infected control vs. PCE treatment: * *p* < 0.05, ** *p* < 0.01, *** *p* < 0.001.

We aimed to identify fungal extracts capable of inhibiting coronavirus replication by assessing the expression levels of viral proteins. RD cells were infected with human coronavirus (HCoV-OC43) and treated with various fungal extracts. Through this screening, we identified one extract exhibiting antiviral activity, and the producing fungus was subsequently identified as *Penicillium compactum*. To further characterize its antiviral effect, we treated coronavirus-infected RD cells with the *Penicillium compactum* extract (PCE) and examined viral protein expression in both cell lysates and conditioned media, with the latter containing a virus released from infected cells. Cells were treated with increasing concentrations of PCE (0, 5, 10, and 20 μg/mL), and equal amounts of cell lysates and media were analyzed by Western blot. PCE treatment reduced the expression of coronavirus proteins in both cells and conditioned media in a dose-dependent manner (Figure 1A,B). The calculated half-maximal inhibitory concentration (IC_50_) values were 15.69 μg/mL for cellular viral proteins and 12.33 μg/mL for viral proteins in the conditioned media.

### 2.2. PCE Treatment Ameliorates the Coronavirus Induced Cytotoxicity

**Figure 2:**
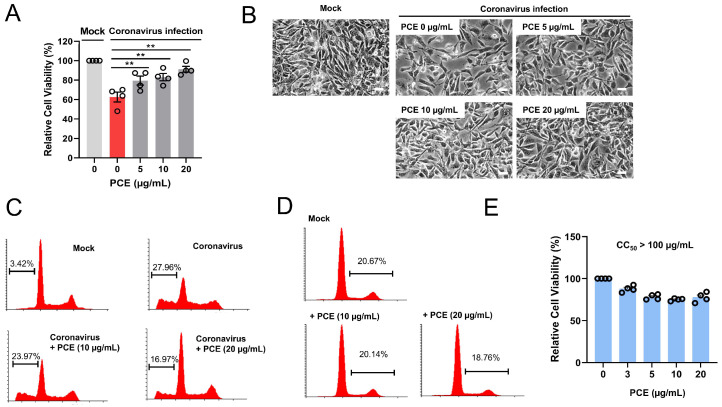
*Penicillium compactum* extract (PCE) ameliorates coronavirus-induced cytotoxicity. (**A**) PCE treatment inhibited coronavirus-induced cell death. RD cells were infected with mock or human coronavirus (HCoV-OC43) and treated with PCE for 72 h. Cell viability was assessed using the MTT assay. (**B**) Representative microscopic images of HCoV-OC43–infected RD cells with or without PCE treatment. Scale bars, 10 µm. (**C**) Flow-cytometric analysis of cell death in HCoV-OC43–infected RD cells treated with PCE. After 72 h of treatment, cells were stained with propidium iodide and analyzed by flow cytometry. (**D**) PCE treatment alone did not induce apoptosis. RD cells were treated with the indicated concentrations of PCE for 72 h and analyzed by flow cytometry. (**E**) PCE treatment resulted in a modest reduction in cell proliferation. RD cells were treated with PCE for 72 h. Cell viability was assessed using the MTT assay. ** *p* < 0.01.

Coronavirus infection induces cytopathic effects in host cells, including cell death. Because PCE treatment reduced coronavirus protein expression, we next investigated whether PCE could also alleviate coronavirus-induced cytotoxicity. RD cells were infected with HCoV-OC43 and treated with PCE. As expected, coronavirus infection led to a marked decrease in cell viability; however, PCE treatment significantly attenuated this cytopathic effect (Figure 2A,B).

Given that PCE improved the viability of infected cells, we further examined apoptotic cell populations using flow cytometry. Consistent with previous findings, coronavirus infection increased the proportion of sub-G1 cells (apoptotic population) compared with mock-treated controls. Notably, PCE treatment reduced the sub-G1 fraction in coronavirus-infected cells (Figure 2C). To determine whether PCE itself induces apoptosis, we quantified the sub-G1 population in cells treated with PCE alone 72 h after treatment. PCE treatment did not increase the proportion of sub-G1 cells, indicating that PCE does not induce apoptosis under these conditions (Figure 2D). We also examined cell proliferation at 72 h after PCE treatment, and found that PCE caused a modest reduction in cell proliferation (Figure 2E). When we attempted to calculate 50% cytotoxicity concentration (CC50), CC50 is well above 100 µM. In addition, S-G2 population is slightly decreased in PCE-treated cells (Figure 2D). Together, these results demonstrate that PCE ameliorates coronavirus-induced cytopathic effects.

### 2.3. PCE Treatment Decreases the Replication of Coronavirus Genomes

**Figure 3:**
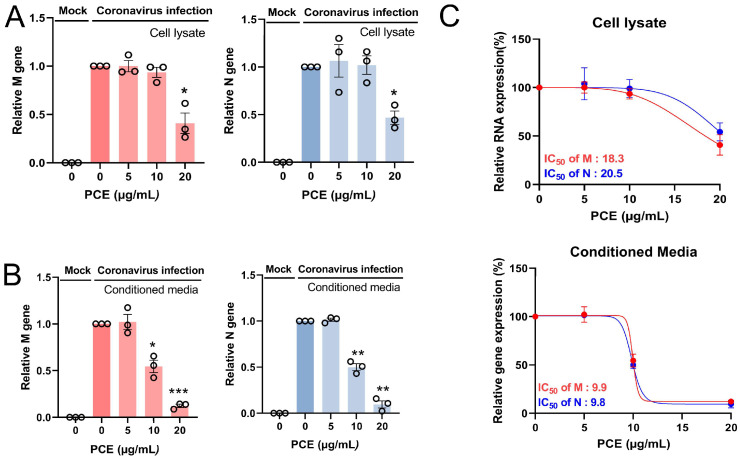
PCE treatment decreases human coronavirus RNA levels. (**A**,**B**) RD cells were infected with human coronavirus (HCoV-OC43) and treated with PCE for 72 h. Total RNA was isolated from the cells (**A**) and conditioned media (**B**), and the levels of viral M and N RNAs were quantified by qRT-PCR. (**C**) The IC_50_ value of PCE was calculated based on viral RNA reduction and is shown in the graph. Infected control vs. PCE treatment: * *p* < 0.05, ** *p* < 0.01, *** *p* < 0.001.

Coronaviruses are RNA viruses; therefore, viral replication can be evaluated by quantifying viral RNA in both cells and conditioned media. Because the viral RNA detected in conditioned media primarily originates from released virion particles, its level reflects the extent of viral production. We measured the RNA levels of the coronavirus membrane (M) and nucleocapsid (N) genes. PCE treatment reduced the expression of both M and N RNAs in a dose-dependent manner in cells and conditioned media (Figure 3A,B). We also calculated the half-maximal inhibitory concentration (IC_50_). The IC_50_ values for intracellular viral RNA were 18.3–20.5 μg/mL, whereas those for viral RNA in conditioned media were 9.8–9.9 μg/mL (Figure 3C). Collectively, these findings demonstrate that PCE treatment inhibits coronavirus replication.

### 2.4. PCE Treatment Decreases Coronavirus-Induced Plaque Formation

**Figure 4:**
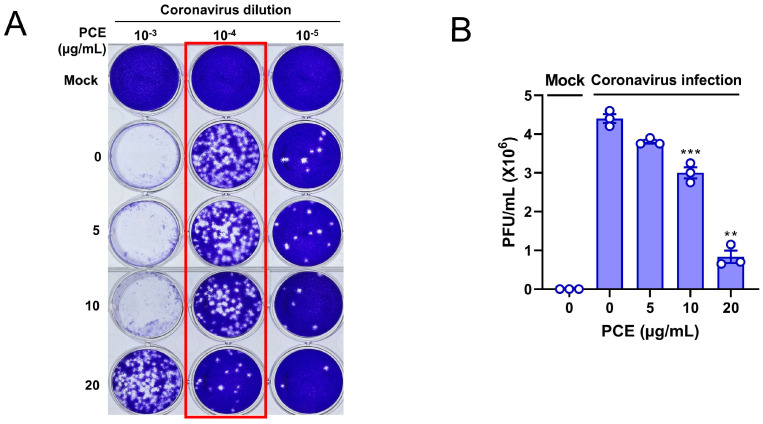
PCE treatment interferes with the production of infectious coronavirus particles. (**A**) PCE treatment reduced the formation of coronavirus-induced plaques. RD cells were infected with human coronavirus (HCoV-OC43) and treated with PCE, followed by plaque assay to assess infectious viral production. (**B**) Plaques were counted, and the quantified values are presented in the graph. Plaque formation assays were performed using three independent biological replicates. Infected control vs. PCE treatment: ** *p* < 0.01, *** *p* < 0.001.

Because PCE treatment inhibited coronavirus replication, we next sought to determine whether it also reduced the production of infectious viral particles. Coronavirus-infected RD cells were treated with the indicated concentrations of PCE, and the resulting conditioned media were used for plaque formation assays. A reduction in infectious virion production would be expected to result in fewer plaques. As anticipated, conditioned media from PCE-treated cells produced markedly fewer plaques, indicating that PCE decreases coronavirus infectivity (Figure 4A). The number of plaques decreased in a dose-dependent manner following PCE treatment (Figure 4B). These results demonstrate that PCE treatment inhibits the production of infectious coronavirus particles.

### 2.5. PCE Treatment Inhibits the Production of Infectious Coronavirus

**Figure 5:**
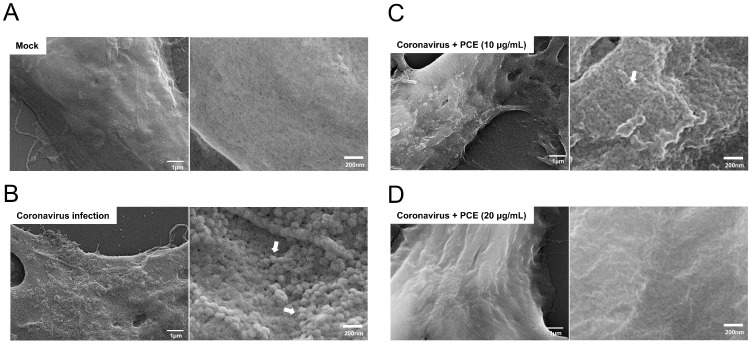
PCE treatment decreases the production of coronavirus virions. (**A**) Mock-treated RD cells exhibited normal cell surface morphology. (**B**) RD cells infected with human coronavirus (HCoV-OC43) showed abundant virion particles on the cell surface. (**C**,**D**) Coronavirus-infected RD cells were treated with the indicated concentrations of PCE for 72 h and analyzed by scanning electron microscopy. PCE treatment reduced the number of virion particles on the cell surface in a dose-dependent manner. The arrow denotes a virion.

Because PCE treatment reduced the level of infectious coronavirus in the conditioned medium, we next sought to visualize its effect on viral production within infected cells. Coronavirus production on the surface of infected cells can be readily observed using scanning electron microscopy (SEM). As expected, coronavirus-infected RD cells exhibited numerous viral particles budding from the cell membrane (Figure 5A,B), with particle sizes of approximately 100 nm, which is consistent with previous reports. When PCE was applied to coronavirus-infected cells, the number of viral particles on the cell surface markedly decreased (Figure 5C,D). At a concentration of 20 μg/mL, PCE nearly eliminated detectable virion production on the plasma membrane. These observations indicate that PCE suppresses coronavirus production in infected cells.

### 2.6. HPLC Analysis of PCE

**Figure 6:**
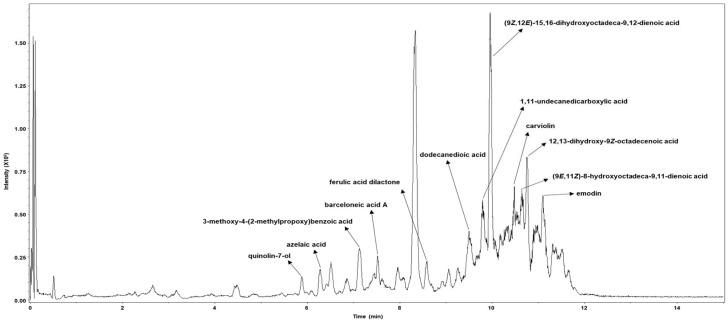
Metabolic profiling of the *Penicillium compactum* extract (PCE). PCE was analyzed using high-performance liquid chromatography (HPLC) coupled with trapped ion mobility spectrometry–time-of-flight mass spectrometry (TIMS-TOF). Individual chromatographic peaks were subjected to MS/MS analysis and annotated based on comparisons with a validated spectral library.

After confirming the antiviral activity of PCE against human coronavirus, we characterized its chemical profile and assessed whether it contains metabolites previously reported to exhibit antiviral activity. Metabolite profiling was performed using HPLC coupled with TIMS-TOF mass spectrometry. A total of 12 individual compounds were identified in PCE, and each chromatographic peak was analyzed and assigned with the corresponding compound (Figure 6, Supplementary Data S1). The identified metabolites were: (1) quinolin-7-ol; (2) azelaic acid; (3) 3-methoxy-4-(2-methylpropoxy) benzoic acid; (4) barceloneic acid A; (5) ferulic acid dilactone; (6) dodecanedioic acid; (7) (9Z,12E)-15,16-dihydroxyoctadeca-9,12-dienoic acid; (8) 1,11-undecanedicarboxylic acid; (9) carviolin; (10) 12,13-dihydroxy-9Z-octadecenoic acid; (11) (9E,11Z)-8-hydroxyoctadeca-9,11-dienoic acid; and (12) emodin.

## 3. Discussion

In this study, we demonstrated that the *Penicillium compactum* extract (PCE) effectively inhibits the replication of human coronavirus. Fungi are well known as prolific producers of bioactive compounds, including antibiotics and other pharmacologically important metabolites. Building on this foundation, we sought to identify fungal strains with previously uncharacterized antiviral properties. Although the COVID-19 pandemic has been mitigated by the development of vaccines and antiviral therapeutics, emerging variants and drug-resistant coronavirus strains continue to pose significant global health concerns. Consequently, the discovery of novel antiviral agents remains an essential and urgent research goal.

Using a human coronavirus (HCoV-OC43) culture system, we screened various fungal extracts for antiviral activity and identified *Penicillium compactum* as a promising candidate. Its extract markedly reduced coronavirus protein expression, prompting further investigation to elucidate its antiviral potential and mechanisms of action.

Our findings indicate that PCE strongly interferes with the coronavirus replication cycle, particularly by affecting late stages of virion assembly and release. The consistently lower IC_50_ values observed in conditioned media compared with cell lysates suggest that PCE exerts a stronger inhibitory effect on extracellular virions than on intracellular viral intermediates. This pattern implies that PCE may target processes involved in virion maturation, assembly, or egress rather than early replication or transcriptional steps.

Multiple lines of evidence support this interpretation. First, both Western blot and quantitative RT-PCR analyses revealed that PCE reduced viral protein and RNA levels more effectively in conditioned media than inside infected cells. Because extracellular viral components primarily originate from released virions, these findings suggest that PCE suppresses virion production or release. Second, plaque assays using conditioned media confirmed a significant reduction in the number of infectious viral particles following PCE treatment, demonstrating that released virions were not only fewer but also less capable of initiating secondary infections. Third, SEM imaging provided direct structural evidence: PCE treatment dramatically decreased the number of coronavirus-like particles on the surface of infected cells, and higher concentrations nearly eliminated detectable virions. The observed particle diameters (~100 nm) were consistent with known coronavirus morphology, reinforcing the validity of these observations.

Collectively, these results suggest that PCE disrupts a late step in the coronavirus life cycle, likely during virion assembly, budding, or membrane-dependent exocytosis. This mechanism distinguishes PCE from many antiviral agents that primarily target viral entry or genome replication. Although the precise molecular targets remain unknown, potential mechanisms include interference with viral envelope formation, disruption of host membrane trafficking pathways, or modulation of lipid metabolic processes required for virion maturation. Future studies integrating proteomic analyses and time-course viral replication experiments will be critical for identifying specific host–virus interactions influenced by PCE.

Metabolite profiling further revealed that PCE contains several compounds with known or predicted antiviral activity (Figure 6). Quinolin derivatives have been proposed as potential inhibitors of coronavirus papain-like protease, while ferulic acid has been reported to inhibit coronavirus 3CL-protease [28,29]. Carviolin has been predicted to interfere with coronavirus receptor binding, and emodin has been shown to inhibit coronavirus 3a protein function [30,31]. These findings suggest that the antiviral activity of PCE may arise from the combined actions of multiple bioactive metabolites.

Overall, our data demonstrate that PCE is a potent inhibitor of coronavirus particle production and release, leading to a substantial reduction in both the number and infectivity of extracellular virions. These results highlight the therapeutic potential of PCE and support further investigation into its active components as candidate antiviral agents targeting the late replicative phase of coronavirus infection.

## 4. Materials and Methods

### 4.1. Strain Cultivation

The fungal strain *Penicillium compactum* was obtained and classified by the Freshwater Bioresources Culture Collection (FBCC), Republic of Korea. The strain was preserved at −80 °C in 15% (*v*/*v*) glycerol stock and revived on potato dextrose agar (PDA; Difco, Detroit, MI, USA). Cultures were incubated at 25 °C for 7 days in an incubator (VS-8480SR, Vision Scientific, Daejeon, Republic of Korea). Three agar plugs (8.75 mm in diameter) were excised from the PDA plate using a No. 4 cork borer, and each plug was aseptically cut into eight equal fragments, yielding a total of 24 agar pieces. These fragments were inoculated into 80 mL of potato dextrose broth (PDB; Difco) in a 250 mL baffled Erlenmeyer flask and incubated at 25 °C with shaking at 150 rpm in a shaking incubator (VS-8480SR, Vision Scientific) for 10 days.

### 4.2. Preparation of Penicillium Compactum Extract

Following cultivation, *Penicillium compactum* cultures were mixed with an equal volume of ethyl acetate and agitated on a rotary shaker at 150 rpm and 28 °C for 3 h. The mixture was then subjected to sonication in an ultrasonic bath for 15 min, repeated twice. After centrifugation, the upper ethyl acetate phase was collected and concentrated to dryness using a rotary evaporator. The resulting extract was weighed and dissolved in DMSO at the desired concentrations for subsequent assays. The final DMSO concentration was 0.2% (*v*/*v*) in all experimental groups (including vehicle control). Mock corresponds to DMSO treatment only.

### 4.3. Infection of Coronavirus

HCoV-OC43 virus was purchased from ATCC (Rockville, MD, USA) and rhabdomyosarcoma (RD) cells were obtained from Korean Cell Line Bank (Seoul, Republic of Korea). RD cells were maintained in DMEM media (Welgene, Seoul, Republic of Korea) supplemented with 10% FBS (Thermo Fisher Scientific, Waltham, MA, USA) and 1% penicillin-streptomycin solution (Welgene, Seoul, Republic of Korea). RD cells were infected with human coronavirus (10^6^ PFU/mL), as described previously [32]. Briefly, cells were incubated with the indicated dilutions of media containing coronavirus (MOI of 0.01), and the infected cells were maintained in MEM media supplemented with 2% FBS. For plaque formation, the conditioned media were collected after 72 h of infection and filtered through 0.45 μm CA membrane filters (Sartorius, Göttingen, Germany). RD cells were incubated in a 12-well plate and infected with the indicated concentration of conditioned media containing coronavirus. The infected cells were incubated at 33 °C for an additional 4 days to allow plaque formation, fixed with 4% paraformaldehyde, and stained with 0.2% crystal violet solution. Cell viability was measured using MTT assay as described previously [33].

### 4.4. Western Blot

The expression levels of coronavirus proteins were evaluated by Western blotting using an anti-HCoV-OC43 antibody. Cells and conditioned media were collected separately and lysed in cell lysis buffer (50 mM NaCl, 50 mM HEPES, pH 7.5, and 1% NP-40) supplemented with a protease inhibitor cocktail. Equal amounts of protein were resolved by SDS-PAGE and transferred onto PVDF membranes (Bio-Rad, Hercules, CA, USA). Membranes were blocked with 3% skim milk in TBS containing 0.1% Tween-20 (TBS-T) and probed with an anti-HCoV-OC43 antibody (Sigma-Aldrich, St. Louis, MO, USA). Immunoreactive bands were visualized using the ChemiDoc Imaging System (Bio-Rad).

### 4.5. Quantitative RT-PCR

Quantitative RT-PCR was performed to measure the levels of coronavirus RNA in both cells and conditioned media. Cells and media were collected separately, and total RNA was extracted using the PURE™ Total RNA Extraction Kit (Infusion Tech, Anyang, Republic of Korea) according to the manufacturer’s instructions. Equal amounts of total RNA were used for cDNA synthesis with the M-MLV cDNA Synthesis Kit (Enzynomics, Seoul, Republic of Korea). qRT-PCR was carried out using the QuantStudio 3 Real-Time PCR System (Thermo Fisher Scientific), and GAPDH was used as an internal control. Primer sequences for viral and host genes were previously described [32].

### 4.6. Flow Cytometry

For cell cycle analysis, RD cells were either mock-treated or infected with human coronavirus and subsequently treated with the indicated concentrations of *Penicillium compactum* extract (PCE). Following incubation, cells were harvested, washed with phosphate-buffered saline (PBS), and fixed in 70% ethanol at 4 °C. Fixed cells were centrifuged, washed once with PBS, and resuspended in PBS containing 50 μg/mL propidium iodide (PI) and 10 mg/mL RNase A (Sigma, St. Louis, MO, USA). DNA content was analyzed using a FACSCalibur flow cytometer (Becton Dickinson, Mountain View, CA, USA), and a minimum of 10,000 events were acquired per sample. Cell cycle distribution was determined using Flowing Software, version 2.5 (Perttu Terho, Turku Centre for Biotechnology, Finland).

### 4.7. Scanning Electron Microscopy (SEM)

For scanning electron microscopy (SEM), RD cells were cultured on sterilized 9-mm coverslips and infected with HCoV-OC43 for 3 days. Cells were fixed with 2.5% glutaraldehyde for 1 h and dehydrated through a graded ethanol series (20%, 40%, 60%, 80%, 90%, and 100%). After dehydration, samples were air-dried in a vacuum desiccator for 1 h. The dried cells were coated with platinum, and SEM images were acquired using a Carl Zeiss SUPRA 40 scanning electron microscope (Carl Zeiss, Oberkochen, Germany).

### 4.8. Metabolite Profiling

Metabolite profiling of *Penicillium compactum* was performed at the Korea Basic Science Institute (KBSI, Seoul, Republic of Korea) using high-performance liquid chromatography (HPLC; 1290 Infinity, Agilent, Palo Alto, CA, USA) coupled with trapped ion mobility spectrometry–time-of-flight mass spectrometry (TIMS-TOF; Bruker, Billerica, MA, USA). The column oven and autosampler were maintained at 25 °C and 4 °C, respectively. Samples were separated on an Acquity UPLC HSS T3 column (1.8 μm, 2.1 mm × 50 mm; Waters, Milford, MA, USA). The mobile phase consisted of 0.1% (*v*/*v*) formic acid and 5 mM ammonium acetate in water (solvent A) and acetonitrile (solvent B). The gradient for solvent B was as follows: 5% at 0.01 min, 35% at 7 min, 60% at 8 min, held until 9 min, returned to initial conditions at 9.1 min, and equilibrated for an additional 3 min at a flow rate of 0.4 mL/min.

Mass spectrometry was conducted over an m/z range of 50–1000 using the following parameters: capillary voltage, 4500 V; end plate offset, 500 V; nebulizer pressure, 2.0 bar; drying gas flow, 10.0 L/min; and source temperature, 220 °C. Data processing, including spectral alignment and peak detection, was performed using MetaboScape 5.0 (Bruker), and compound identification was carried out using the Bruker NIST 2020 MS/MS spectral library.

### 4.9. Statistical Analysis

The results of Western blot, quantitative RT-PCR, and MTT assays were analyzed using two-tailed Student’s *t*-tests in Excel (Microsoft, Redmond, WA, USA). IC_50_ values were calculated, and bar graphs were generated using GraphPad Prism 8 (GraphPad Software, CA, USA). A *p*-value of <0.05 was considered statistically significant.

## 5. Conclusions

Although vaccines and antiviral agents against human coronaviruses have been developed, alternative strategies are still required to counter the emergence of diverse viral variants. Fungal extracts are known to exert a wide range of biological activities, and in this study, we demonstrated that the extract from *Penicillium compactum* effectively inhibits the replication of human coronavirus. These findings suggest that *Penicillium compactum* extract may serve as a promising alternative approach for the treatment of coronavirus infections.

## Data Availability

The original contributions presented in this study are included in the article. Further inquiries can be directed to the corresponding authors.

## Supplementary Materials

The following supporting information can be downloaded at: https://www.mdpi.com/article/10.3390/ijms27031183/s1.
