# Supplementary material for: The Extract of the Endophytic Fungus Penicillium compactum Inhibits the Replication of Human Coronavirus
Source: Int J Mol Sci. 2026 Jan 24;27(3):1183. doi: 10.3390/ijms27031183 (PMC12897886; doi:10.3390/ijms27031183)
Supplement: Supplementary file 1 [file ijms-27-01183-s001.zip › ijms-4033476-supplementary.pdf]

Supplementary Data S1. List of metabolites from PCE.

No.	Name	Intensity
1	Quinolin-7-ol	5354.86 ± 407.38
2	Azelaic acid	7923.57 ± 311.63
3	3-Methoxy-4-(2-methylpropoxy)benzoic acid	16644.43 ± 369.84
4	Barceloneic acid A	167026.29 ± 5093.74
5	Ferulic acid dilactone	1035.86 ± 92.78
6	Dodecanedioic acid	3570.29 ± 309.23
7	1,11-Undecanedicarboxylic acid	1061.14 ± 125.32
8	(9Z,12E)-15,16-Dihydroxyoctadeca-9,12-dienoic acid	19031.57 ± 759.02
9	Carviolin	6240.86 ± 237.34
10	(9E,11Z)-8-Hydroxyoctadeca-9,11-dienoic acid	38361.86 ± 5114.63
11	12,13-Dihydroxy-9Z-octadecenoic acid	5812.43 ± 1160.43
12	Emodin	18609.71 ± 1430.26
